# Targeting peroxisomal fatty acid oxidation improves hepatic steatosis and insulin resistance in obese mice

**DOI:** 10.1016/j.jbc.2022.102845

**Published:** 2022-12-28

**Authors:** Haoya Yao, Yaoqing Wang, Xiao Zhang, Ping Li, Lin Shang, Xiaocui Chen, Jia Zeng

**Affiliations:** School of Life Science, Hunan University of Science and Technology, Xiangtan, Hunan, PR China

**Keywords:** fatty acid oxidation, peroxisomes, mitochondria, acetyl-carnitine, obesity, ACD, acyl-CoA dehydrogenase, CAT, carnitine acetyltransferase, FAO, fatty acid oxidation, HOMA-IR, homeostasis model assessment of insulin resistance, IR, insulin resistance, LCACD, long-chain acyl-CoA dehydrogenase, LC-CoA, long-chain acyl-CoA, MCACD, medium-chain acyl-CoA dehydrogenase, TAG, triacylglyceride, TDYA, 10,12-tricosadiynoic acid

## Abstract

Obesity and diabetes normally cause mitochondrial dysfunction and hepatic lipid accumulation, while fatty acid synthesis is suppressed and malonyl-CoA is depleted in the liver of severe obese or diabetic animals. Therefore, a negative regulatory mechanism might work for the control of mitochondrial fatty acid metabolism that is independent of malonyl-CoA in the diabetic animals. As mitochondrial β-oxidation is controlled by the acetyl-CoA/CoA ratio, and the acetyl-CoA generated in peroxisomal β-oxidation could be transported into mitochondria via carnitine shuttles, we hypothesize that peroxisomal β-oxidation might play a role in regulating mitochondrial fatty acid oxidation and inducing hepatic steatosis under the condition of obesity or diabetes. This study reveals a novel mechanism by which peroxisomal β-oxidation controls mitochondrial fatty acid oxidation in diabetic animals. We determined that excessive oxidation of fatty acids by peroxisomes generates considerable acetyl-carnitine in the liver of diabetic mice, which significantly elevates the mitochondrial acetyl-CoA/CoA ratio and causes feedback suppression of mitochondrial β-oxidation. Additionally, we found that specific suppression of peroxisomal β-oxidation enhances mitochondrial fatty acid oxidation by reducing acetyl-carnitine formation in the liver of obese mice. In conclusion, we suggest that induction of peroxisomal fatty acid oxidation serves as a mechanism for diabetes-induced hepatic lipid accumulation. Targeting peroxisomal β-oxidation might be a promising pathway in improving hepatic steatosis and insulin resistance as induced by obesity or diabetes.

The mechanism for the control of mitochondrial fatty acid oxidation (FAO) by carbohydrate was established 40 years ago, and malonyl-CoA was identified to be a key molecule for the regulation of mitochondrial fatty acids oxidation (1, 2). However, it is striking to note that under the condition of severe obesity or diabetes when the supply of glucose is limited, fatty acid biosynthesis is suppressed and liver malonyl-CoA is depleted (3, 4, 5), while significant lipid accumulation is observed in the liver of diabetic animals as well as patients (6, 7, 8, 9). Therefore, a putative regulatory mechanism might work for the restriction of excessive fatty acids burning in mitochondria that is independent of malonyl-CoA in the liver of obese or diabetic animals.

To explore the potential mechanism for diabetes-induced mitochondrial dysfunction and hepatic steatosis, we shed light on peroxisomal β-oxidation, an FAO system that metabolizes long-chain and branched chain fatty acids (10, 11). Previous studies have provided evidence that peroxisomal β-oxidation plays a negative role in regulating mitochondrial FAO by increasing biosynthesis of malonyl-CoA in rats-fed high fat diet or elevating mitochondrial NADH redox state in the fasting animals (12, 13). Although the crosstalk between peroxisomal β-oxidation and mitochondrial FAO in diabetic animals is not established so far, we noted the well-known fact that mitochondrial FAO is controlled by intramitochondrial acetyl-CoA/CoA ratio, and elevation in this ratio might cause suppression of mitochondrial 3-ketoacyl-CoA thiolase and accumulation of reaction intermediates (14, 15), which results in feedback inhibition of mitochondrial β-oxidation (16, 17). As peroxisomal β-oxidation is induced in the liver of diabetic animals (18, 19, 20), the acetyl-CoA generated in peroxisomal β-oxidation can be transported into mitochondria via carnitine shuttles (21, 22, 23), which has the potential to mediate mitochondrial acetyl-CoA/CoA ratio. We hypothesized that peroxisomal oxidation of endogenous fatty acids might control mitochondrial β-oxidation and inducing hepatic steatosis in the obese or diabetic animals.

This study investigated the role and potential mechanism of peroxisomal β-oxidation in regulating mitochondrial FAO in the liver of *ob/ob* obese mice.

## Results

### Peroxisomal β-oxidation was induced in the liver of ob/ob mice

**Figure 1:**
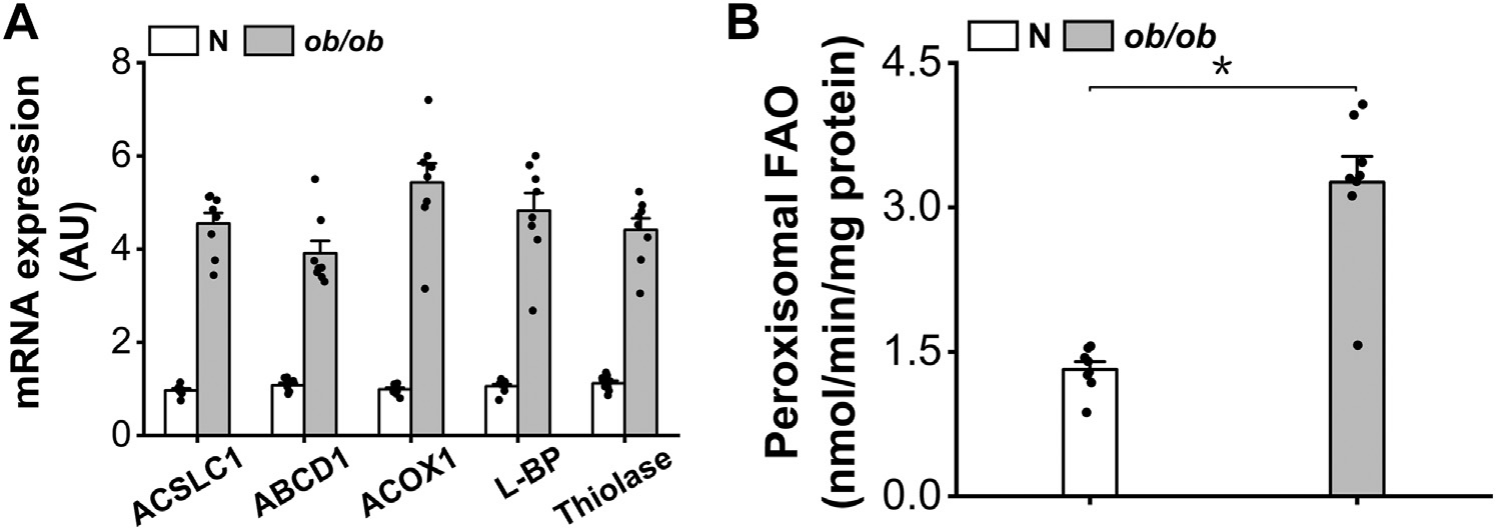
**Peroxisomal β-oxidation is induced in the liver of *ob/ob* mice.***A*, gene expressions of the enzymes involved in peroxisomal β-oxidation were induced in the liver of *ob/ob* mice. *B*, peroxisomal β-oxidation increased remarkably in the liver of *ob/ob* mice. ∗*p* < 0.05 by *t* test between paired groups.

As a typical animal model of obesity and type 2 diabetes, *ob/ob* mice was used for the study of peroxisomal β-oxidation in regulating mitochondrial FAO. Elevation in plasma-free fatty acid in *ob/ob* mice results in the increased uptake of fatty acids in liver and activation of PPARα (24), as reflected by upregulation in the gene expressions of the enzymes involved in peroxisomal β-oxidation in the liver of *ob/ob* mice, as shown in Figure 1*A*. The activity of peroxisomal β-oxidation increased significantly in the liver of *ob/ob* mice (Fig. 1*B*).

### Suppression of peroxisomal β-oxidation improves hepatic steatosis and insulin resistance in ob/ob mice

**Figure 2:**
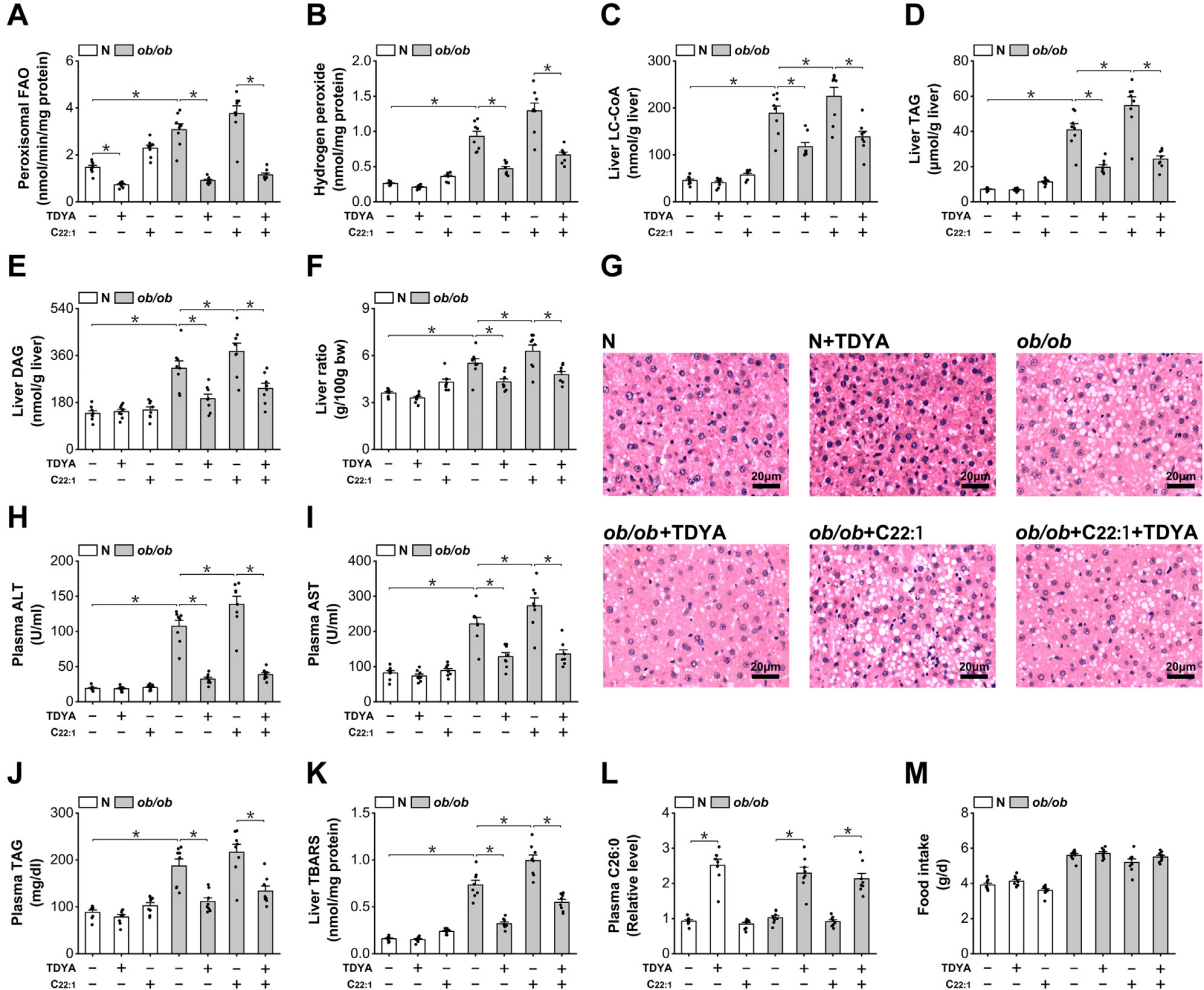
**Suppression of peroxisomal β-oxidation improves hepatic steatosis in *ob/ob* mice.***A*, peroxisomal β-oxidation in the liver of *ob/ob* mice was suppressed after treatment with TDYA. *B*, TDYA treatment significantly reduced hydrogen peroxide formation in the liver of *ob/ob* mice. *C*, LC-CoA increased significantly in the liver of *ob/ob* mice, which was reduced by the treatment of TDYA. *D*, liver TAG was significantly higher in the liver of *ob/ob* mice, as further increased by the treatment of C22:1 and reduced by TDYA. *E*, liver DAG was significantly higher in the liver of *ob/ob* mice, as further increased by the treatment of C22:1 and reduced by TDYA. *F*, C22:1 treatment significantly elevated the liver ratio of *ob/ob* mice, as lowered by the treatment of TDYA. *G*, liver histologic changes in *ob/ob* mice treated with C22:1 and TDYA. *H* and *I*, plasma (H) ALT and (I) AST were elevated remarkably in *ob/ob* mice compared to the normal group, as lowered by the treatment of TDYA. *J*, plasma TAG was significantly higher in *ob/ob* mice than in normal group, as reduced by the treatment with TDYA. *K*, TBARS increased significantly in the liver of *ob/ob* mice, as further increased by the treatment of C22:1 and reduced by TDYA. *L*, TDYA treatment caused significant elevation in plasma C26:0 in *ob/ob* mice. *M*, daily food intake was not affected in *ob/ob* mice treated with TDYA or C22:1. ∗*p* < 0.05 by *t* test between paired groups. TDYA, 10,12-tricosadiynoic acid; LC-CoA, long-chain acyl-CoA; TAG, triacylglyceride; DAG, diacylglyceride; ALT, alanine aminotransferase; AST, aspartate aminotransferase; TBARS, Thiobarbituric acid-reactive substances.

To investigate whether peroxisomal β-oxidation might play a role in regulating mitochondria FAO and inducing hepatic steatosis in the obese and diabetic animals, we used erucic acid (C22:1), a very long-chain fatty acid to specifically induce peroxisomal β-oxidation flux (25), and 10,12-tricosadiynoic acid (TDYA), a specific inhibitor for acyl-CoA oxidase-1 (ACOX1) to suppress peroxisomal β-oxidation (26). Peroxisomal β-oxidation was strongly suppressed in the liver of *ob/ob* mice after treatment with TDYA (Fig. 2*A*). Hydrogen peroxide, a byproduct of peroxisomal β-oxidation, increased significantly in the liver of *ob/ob* mice; administration of C22:1 led to further increase in hydrogen peroxide in *ob/ob* mice, which was reduced by the treatment of TDYA. Liver long-chain acyl-CoA (LC-CoA), triacylglyceride (TAG), and diacylglyceride were significantly higher in the o*b/ob* mice than the normal mice, as further increased after treatment with C22:1 and reduced by the treatment of TDYA, as shown in Figure 2, *C*–*E*. Accumulation of lipids led to significant increase in liver ratios as well as hepatic steatosis in *ob/ob* mice as reflected by liver sections, while C22:1 treatment exacerbated hepatic steatosis in *ob/ob* mice, and suppression of peroxisomal β-oxidation by TDYA improved hepatic steatosis in *ob/ob* mice (Fig. 2, *F* and *G*). We further determined plasma alanine aminotransferase and aspartate aminotransferase which are measures of hepatic steatosis; the results indicated that plasma aspartate aminotransferase and alanine aminotransferase were significantly higher in *ob/ob* mice than the normal mice, as were further elevated in the *ob/ob* mice treated with C22:1 and lowered by TDYA (Fig. 2, *H* and *I*). C22:1 treatment also caused increase in plasma TAG in *ob/ob* mice and lowered by the treatment of TDYA (Fig. 2*J*). Thiobarbituric acid-reactive substances as an index for oxidative stress increased significantly in the liver of *ob/ob* mice, as further increased after C22:1 feeding and reduced by TDYA, as shown in Figure 2*K*. Peroxisomal β-oxidation plays a critical role in the metabolism of very long-chain fatty acids; block of peroxisomal β-oxidation in ACOX1-/- mice results in the accumulation of very long-chain fatty acids (11). Plasma levels of hexacosanoic acid (C26:0) were then measured by GC-MS and as expected, TDYA treatment caused significant elevation in plasma C26:0 in C57BL/6J and *ob/ob* mice, as shown in Figure 2*L*. Daily food intake was not affected in *ob/ob* mice treated with TDYA or C22:1 (Fig. 2*M*).

**Figure 3:**
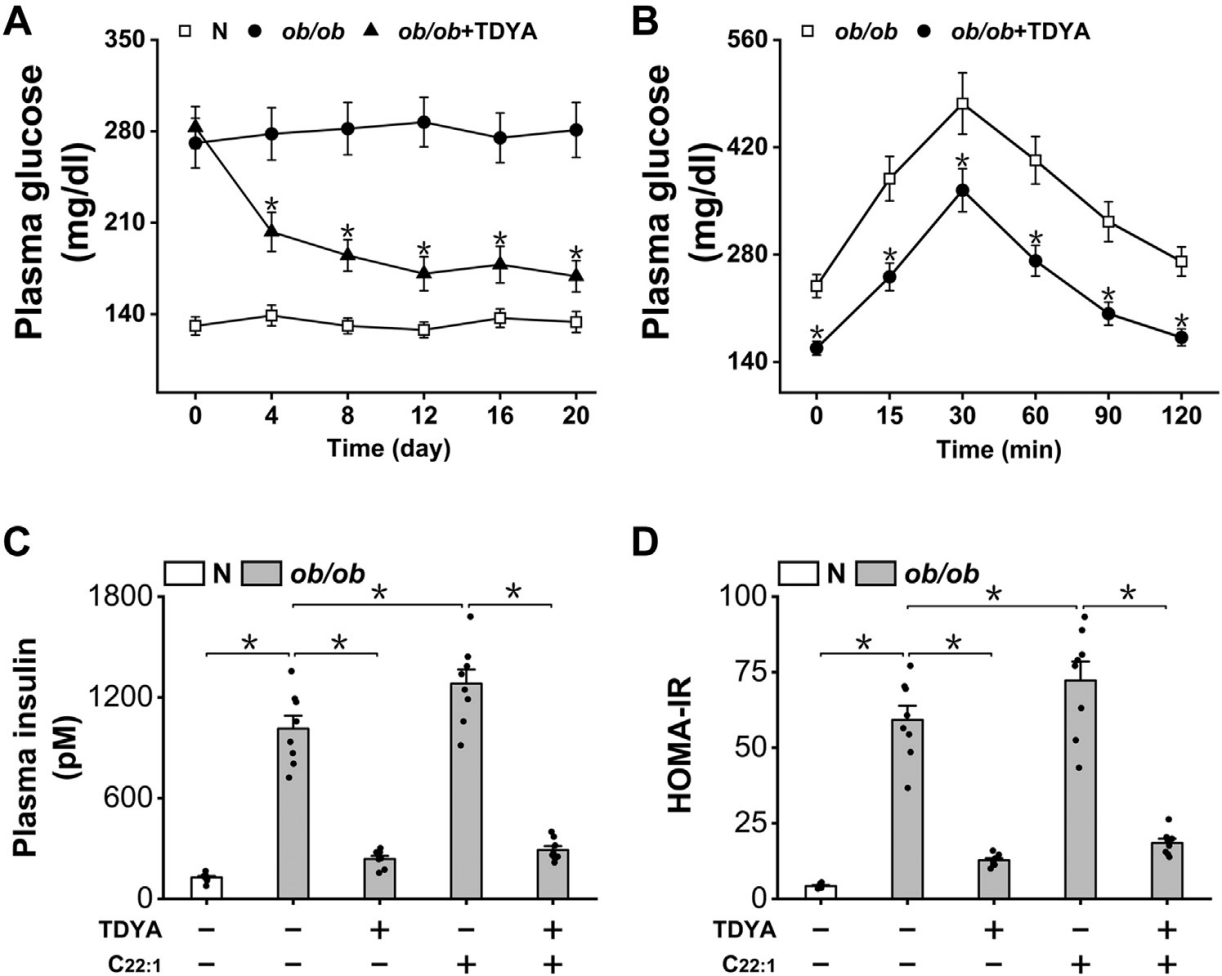
**Suppression of peroxisomal β-oxidation improves insulin resistance in *ob/ob* mice.***A*, TDYA treatment significantly lowered plasma glucose of *ob/ob* mice. *B*, OGTT was improved in *ob/ob* mice treated with TDYA. *C*, plasma insulin increased remarkably in *ob/ob* mice, as lowered after treatment with TDYA. *D*, HOMA-IR was significantly higher in *ob/ob* mice, as further enhanced by the treatment of C22:1 and lowered by TDYA. ∗*p* < 0.05 by *t* test between paired groups. TDYA, 10,12-tricosadiynoic acid; IR, insulin resistance; HOMA-IR, homeostasis model assessment of insulin resistance; OGTT, oral glucose tolerance test.

It has been well accepted that accumulation of lipids plays a critical role in inducing insulin resistance (IR) (27, 28, 29). As suppression of peroxisomal β-oxidation significantly reduced haptic lipid and ROS level, we proposed that targeting peroxisomal β-oxidation might improve IR and decrease plasma glucose. Plasma glucose and homeostasis model assessment of insulin resistance (HOMA-IR) index was then measured to determine whether suppression of peroxisomal β-oxidation might improve IR and affect glucose homeostasis in *ob/ob* mice; the results indicated that administration of TDYA significantly lowered plasma glucose in *ob/ob* diabetic mice (Fig. 3*A*). Plasma insulin and HOMA-IR were also significantly lower in *ob/ob* mice treated with TDYA, while C22:1 feeding caused further increase in plasma insulin and HOMA-IR in *ob/ob* mice, as shown in Figure 3, *B* and *C*.

The results suggested that excessive substrate flux through peroxisomal β-oxidation induced lipid accumulation in *ob/ob* diabetic mice, while specific suppression of peroxisomal β-oxidation improved hepatic steatosis and IR in *ob/ob* diabetic mice.

### Induction of peroxisomal β-oxidation suppressed mitochondrial β-oxidation in ob/ob mice independent of malonyl-CoA

**Figure 4:**
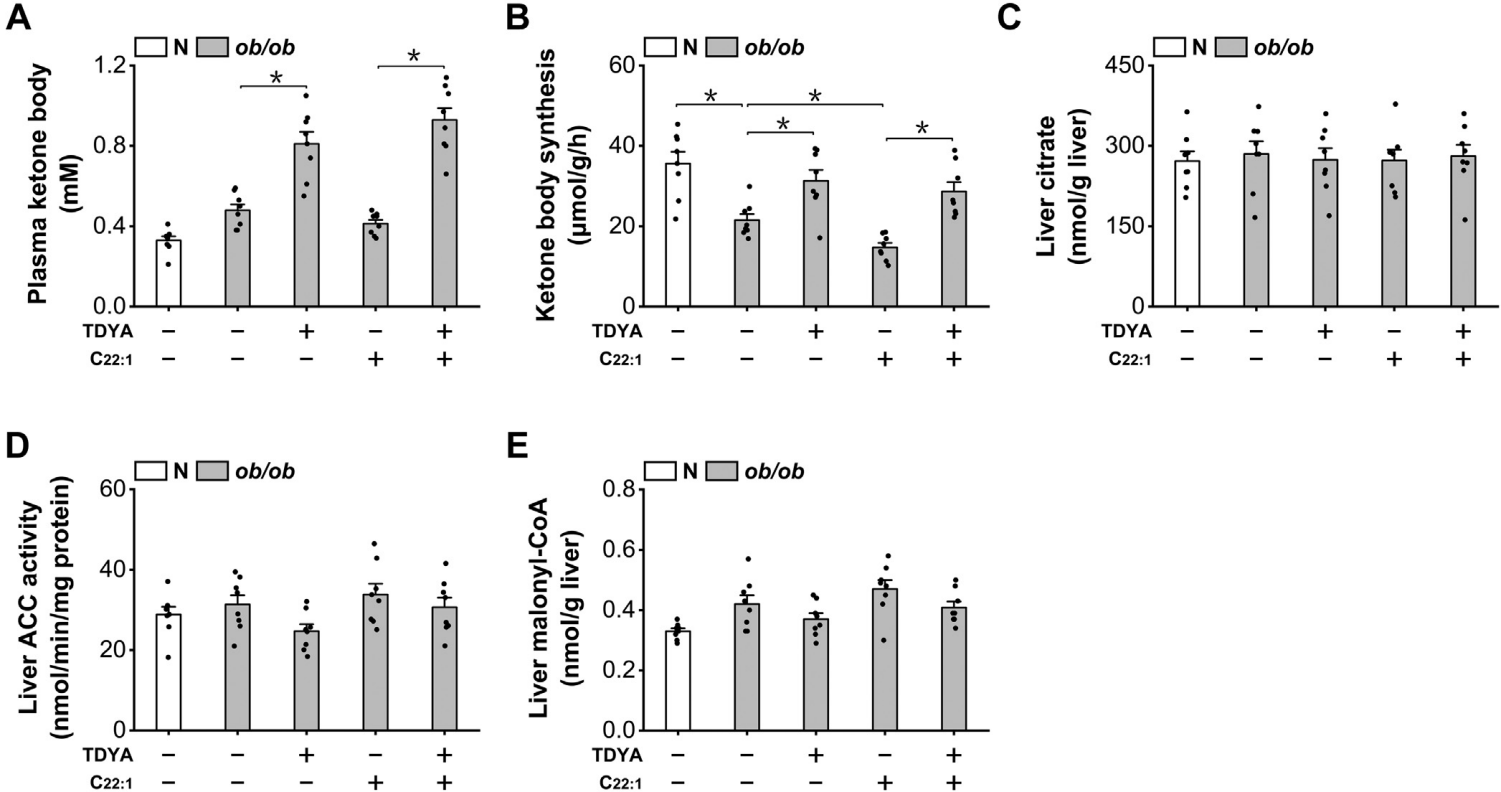
**Induction of peroxisomal β-oxidation suppresses mitochondrial FAO independent of malonyl-CoA.***A*, C22:1 feeding lowered and TDYA treatment elevated plasma ketone body in *ob/ob* mice. *B*, ketone body synthesis rate was significantly lower in *ob/ob* mice than the normal group, as further decreased by C22:1 feeding and recovered by TDYA. *C*, liver citrate content was not altered significantly among all the groups. *D*, liver ACC activity was not altered among all the groups. *E*, liver malonyl-CoA was not significantly changed in *ob/ob* mice treated with C22:1 or TDYA. ∗*p* < 0.05 by *t* test between paired groups. FAO, fatty acid oxidation; TDYA, 10,12-tricosadiynoic acid; ACC, acetyl-CoA carboxylase.

To investigate the potential mechanism by which peroxisomal β-oxidation induces hepatic steatosis in *ob/ob* mice, we shed light on mitochondrial FAO as mitochondrial β-oxidation plays a central role in liver lipid homeostasis. Plasma ketone body and ketone body synthesis rate as measures of mitochondrial FAO were then determined; the results suggested that administration of TDYA significantly increased plasma ketone body level and ketone body synthesis rate in *ob/ob* mice, which were lowered by the treatment of C22:1, as shown in Figure 4, *A* and *B*. Therefore, suppression of peroxisomal β-oxidation stimulated mitochondrial FAO in the diabetic mice. Liver citrate and acetyl-CoA carboxylase, the key enzyme in malonyl-CoA formation were not significantly altered among all the groups (Fig. 4, *C* and *D*). Malonyl-CoA, a core molecule in regulating mitochondrial FAO was also not significantly changed after treatment with C22:1 or TDYA in *ob/ob* mice, as shown in Figure 4*E*. The results suggested that induction of peroxisomal β-oxidation caused suppression of mitochondrial FAO in the liver of *ob/ob* mice independent of malonyl-CoA.

### Peroxisomal β-oxidation increased mitochondrial acetyl-CoA/CoA ratio and suppressed ACD activity

**Figure 5:**
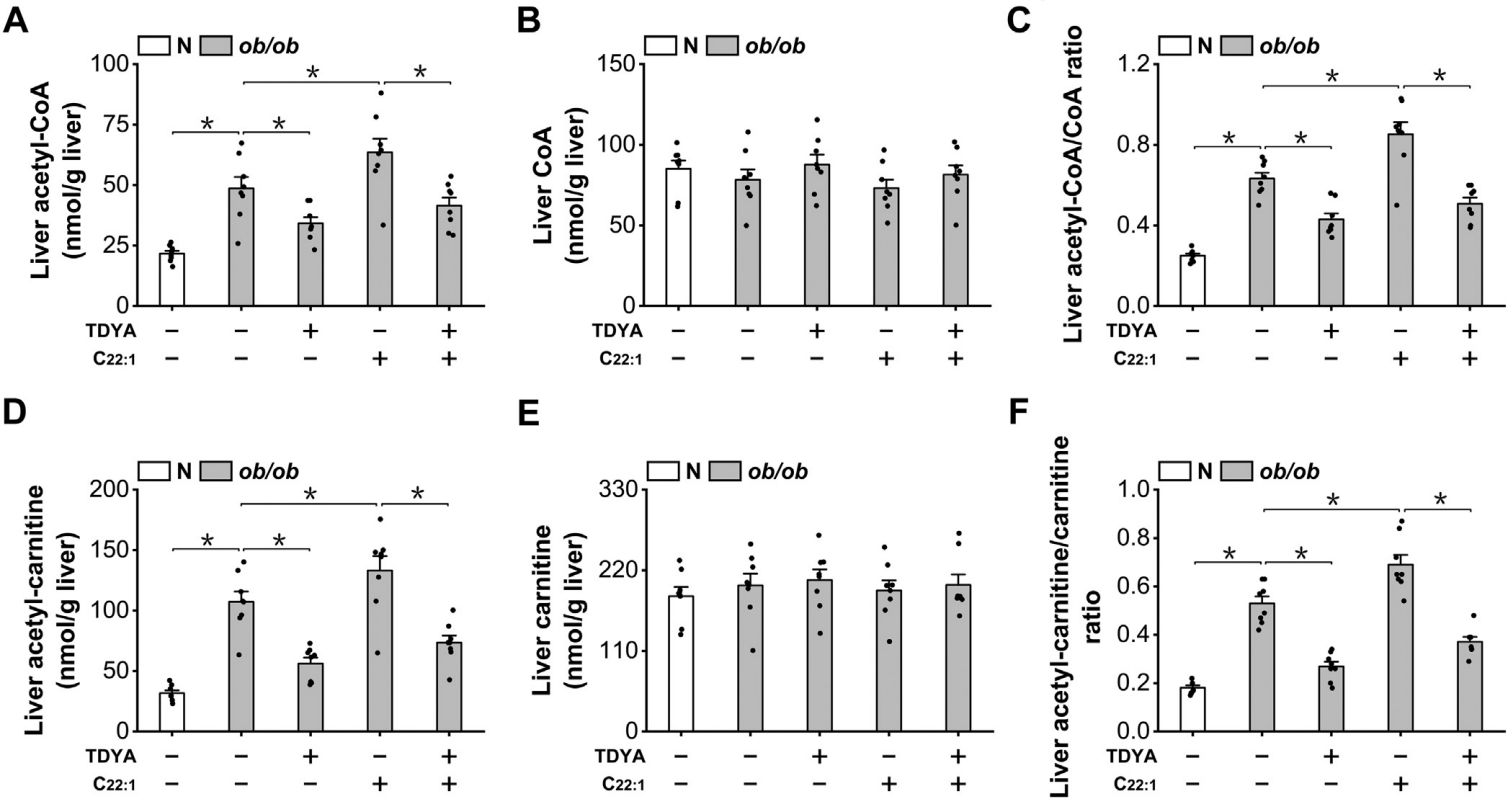
**Peroxisomal β-oxidation of fatty acids increases mitochondrial acetyl-CoA/CoA ratio.***A*, liver acetyl-CoA increased considerably in the liver of *ob/ob* mice, and C22:1 feeding caused further increase in mitochondrial acetyl-CoA, which was lowered by TDYA. *B*, liver free CoA was not altered significantly among all the groups. *C*, liver acetyl-CoA/CoA ratio was elevated significantly in *ob/ob* mice compared to normal mice, as further enhanced by C22:1 feeding and lowered by TDYA. *D*, liver acetyl-carnitine increased significantly in *ob/ob* mice compared to the normal mice, and C22:1 feeding caused further increase in acetyl-carnitine level, which was reduced by TDYA. *E*, liver free carnitine levels were not significantly changed after treatment of C22:1 or TDYA. *F*, liver acetyl-carnitine/carnitine ratio was elevated significantly in *ob/ob* mice compared to normal mice, as further enhanced by C22:1 feeding and lowered by TDYA. ∗*p* < 0.05 by *t* test between paired groups. TDYA, 10,12-tricosadiynoic acid.

To explore the mechanism by which peroxisomal β-oxidation regulates mitochondrial FAO in the liver of diabetic mice, we noted that mitochondrial β-oxidation is under the control of acetyl-CoA/CoA ratio, elevation in this ratio causes suppression of mitochondrial FAO (14, 15, 16, 17), and the acetyl-CoA generated in peroxisomal β-oxidation could be transformed to mitochondria via carnitine shuttles (21, 22, 23). Therefore, peroxisomal β-oxidation might play a role in controlling mitochondrial FAO through mediating mitochondrial acetyl-CoA/CoA ratio. Liver acetyl-CoA was measured and the results indicated that the acetyl-CoA in liver increased considerably in *ob/ob* mice compared to normal mice; C22:1 treatment caused further increase in acetyl-CoA while TDYA treatment significantly lowered liver acetyl-CoA level (Fig. 5*A*). Liver free CoA was not changed significantly among all the groups (Fig. 5*B*). Liver acetyl-CoA/CoA ratio was elevated significantly in *ob/ob* mice compared to normal mice, as further elevated by C22:1 feeding and lowered by TDYA, as shown in Figure 5*C*.

The measured acetyl-CoA/CoA ratio in liver homogenate could partly represent intramitochondrial ratio of acetyl-CoA/CoA. To obtain more evidence on mitochondrial ratio of acetyl-CoA/CoA, we note the fact that intramitochondrial acetyl-CoA/CoA ratio is in equilibrium with acetyl-carnitine/carnitine ratio, and increased generation of acetyl-carnitine causes elevation in mitochondrial acetyl-CoA/CoA ratio (16, 30). Liver acetyl-carnitine was then measured and the results indicated that acetyl-carnitine increased significantly in the liver of *ob/ob* mice compared to the normal mice; TDYA treatment significantly reduced acetyl-carnitine level and C22:1 treatment led to a further increase in acetyl-carnitine level in *ob/ob* mice (Fig. 5*D*). Liver free carnitine level was not significantly changed after treatment of C22:1 or TDYA, as shown in Figure 5*E*. Liver acetyl-carnitine/carnitine ratio was elevated significantly in *ob/ob* mice compared to normal mice, as further elevated by C22:1 feeding and lowered by TDYA, as shown in Figure 5*F*. The results provided evidence that mitochondrial acetyl-CoA/CoA ratio increased significantly in the liver of *ob/ob* diabetic mice, as lowered by TDYA, a specific inhibitor of peroxisomal β-oxidation.

**Figure 6:**
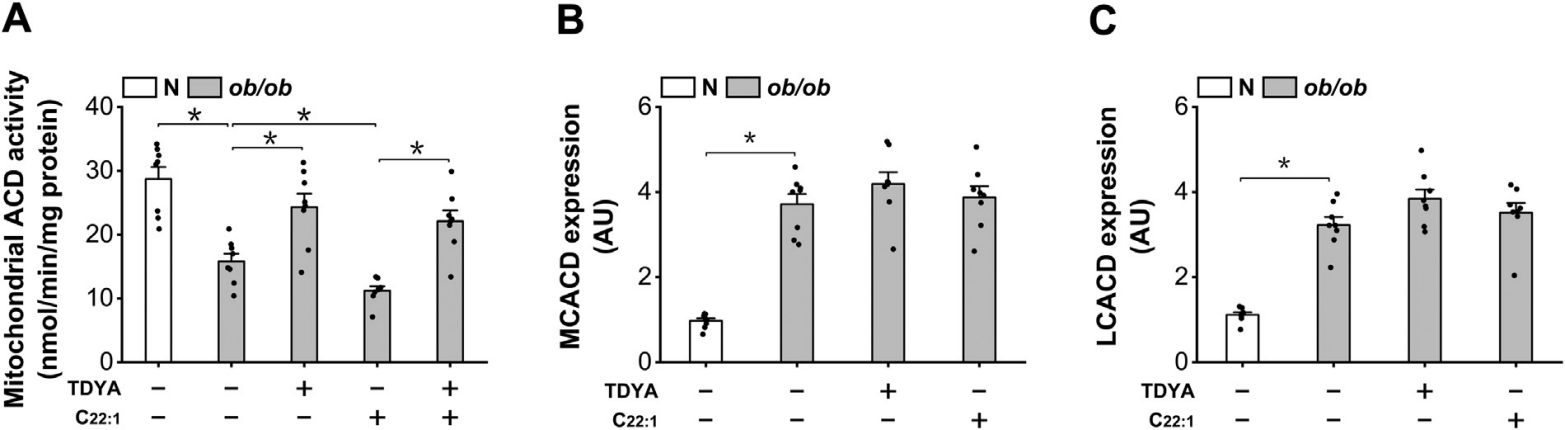
**Induction of peroxisomal β-oxidation causes feedback suppression of mitochondrial acyl-CoA dehydrogenase.***A*, the activity of ACD in intact mitochondria was diminished in *ob/ob* mice, as recovered after treatment with TDYA. *B* and *C*, C22:1 or TDYA treatment did not cause significant changes in the mRNA expressions of (*B*) MCACD and (*C*) LCACD in the liver of *ob/ob* mice. ∗*p* < 0.05 by *t* test between paired groups. TDYA, 10,12-tricosadiynoic acid; ACD, acyl-CoA dehydrogenase; MCACD, medium-chain acyl-CoA dehydrogenase; LCACD, long-chain acyl-CoA dehydrogenase.

Elevation in mitochondrial acetyl-CoA/CoA ratio has been well known to suppress 3-ketoacyl-CoA thiolase activity and further cause feedback suppression of mitochondrial β-oxidation flux at the step of acyl-CoA dehydrogenase (ACD), the rate-limiting enzyme in mitochondrial β-oxidation (31, 32). Mitochondrial ACD was measured and the results indicated that the activity of ACD in intact mitochondria was diminished in *ob/ob* mice, while TDYA significantly increased mitochondrial ACD activity, as shown in Figure 6*A*. mRNA expressions of medium-chain acyl-CoA dehydrogenase (MCACD) and long-chain acyl-CoA dehydrogenase (LCACD) were also analyzed; C22:1 or TDYA treatment did not cause significant changes in the expression of the MCACD and LCACD in the liver of *ob/ob* mice (Fig. 6, *B* and *C*), indicating enzyme expressions were not involved in the control mechanism.

The results suggested that mitochondrial FAO could be regulated by peroxisomal β-oxidation through alteration in mitochondrial acetyl-CoA/CoA ratio.

### Peroxisomal β-oxidation increased generation of acetyl-carnitine

**Figure 7:**
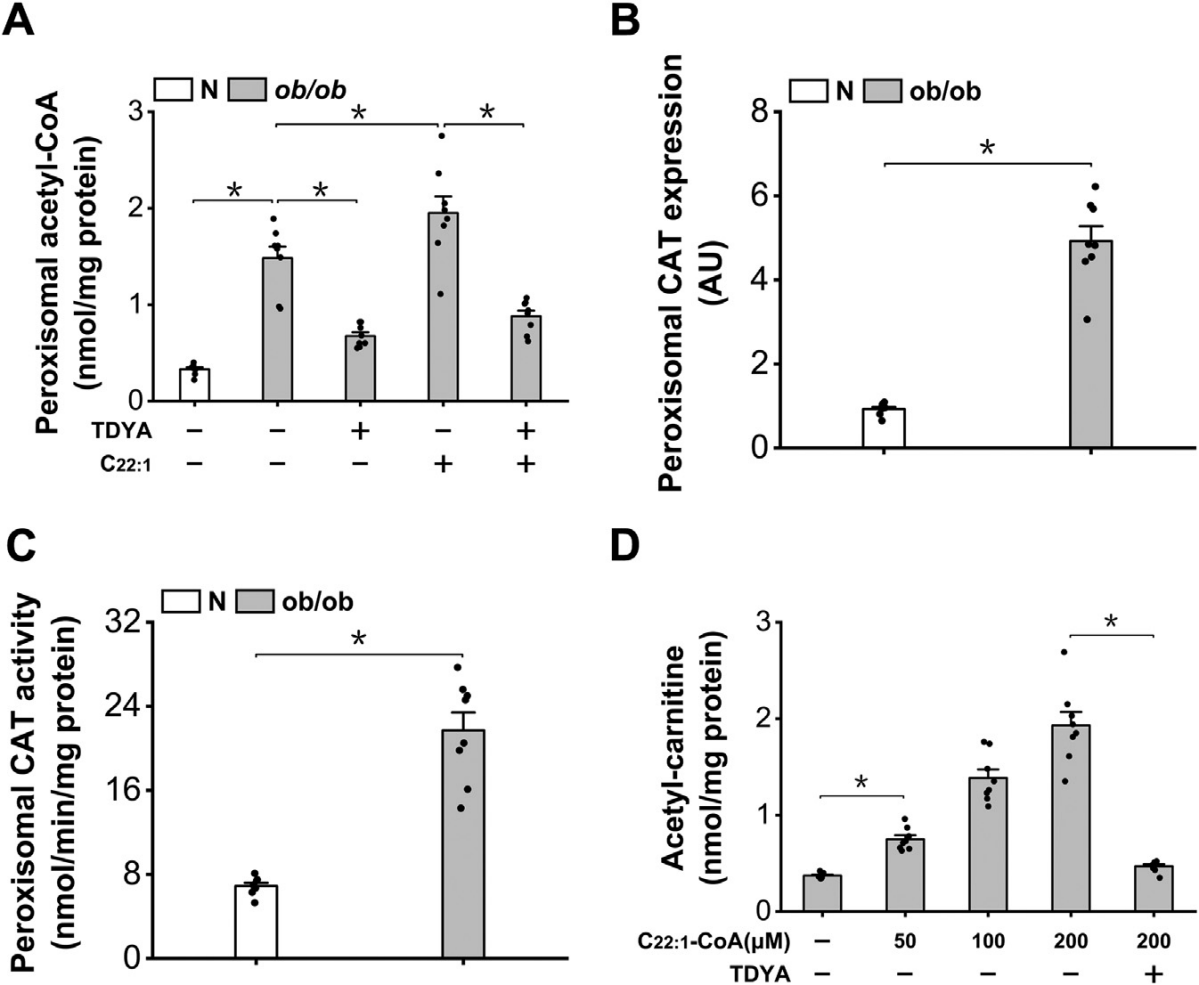
**Peroxisomal β-oxidation generates acetyl-carnitine.***A*, the content of peroxisomal acetyl-CoA increased significantly in the liver of *ob/ob* mice and reduced after treatment with TDYA. *B*, mRNA expression of peroxisomal CAT increased remarkably in the liver of *ob/ob* mice compared to the normal control. *C*, peroxisomal CAT activity increased significantly in the liver of *ob/ob* mice. *D*, addition of erucyl-CoA (C22:1-CoA) to the isolated peroxisomes from the liver of *ob/ob* mice generated acetyl-carnitine dose-dependently, as suppressed by pretreatment with TDYA. ∗*p* < 0.05 by *t* test between paired groups. TDYA, 10,12-tricosadiynoic acid; CAT, carnitine acetyltransferase.

It is well known that the acetyl-CoA generated from peroxisomal β-oxidation can be transformed to acetyl-carnitine via peroxisomal carnitine acetyltransferase (CAT) (11, 21, 22, 23). Therefore, induction of peroxisomal β-oxidation might increase the supply of acetyl-carnitine that regulates mitochondrial acetyl-CoA level. To confirm that the increased acetyl-carnitine that caused suppression of mitochondrial FAO was originated from peroxisomal β-oxidation, peroxisomal acetyl-CoA was analyzed and the results suggested that peroxisomal acetyl-CoA increased remarkably in the liver of *ob/ob* mice, as further elevated after treatment with C22:1 and reduced by TDYA (Fig. 7*A*). In the meantime, the mRNA expression level and activity of peroxisomal CAT increased significantly in the liver of *ob/ob* mice (Fig. 7, *B* and *C*), which accelerated the formation of acetyl-carnitine from acetyl-CoA generated in peroxisomal β-oxidation. Formation of acetyl-carnitine by peroxisomal β-oxidation was further confirmed by incubating erucyl-CoA (22:1-CoA) with isolated peroxisomes from mouse liver. Addition of C22:1-CoA to the isolated peroxisomes led to dose-dependent generation of acetyl-carnitine, as suppressed by pretreatment of TDYA-CoA, and peroxisomes from the liver of *ob/ob* mice showed much higher capacity in the generation of acetyl-carnitine than normal mice, as shown in Figure 7*D*. The results supported that peroxisomal β-oxidation increased the supply of acetyl-carnitine that controlled liver acetyl-CoA/CoA ratio and β-oxidation, and suppression of peroxisomal β-oxidation enhanced mitochondrial FAO by reducing acetyl-carnitine generation.

## Discussion

**Figure 8:**
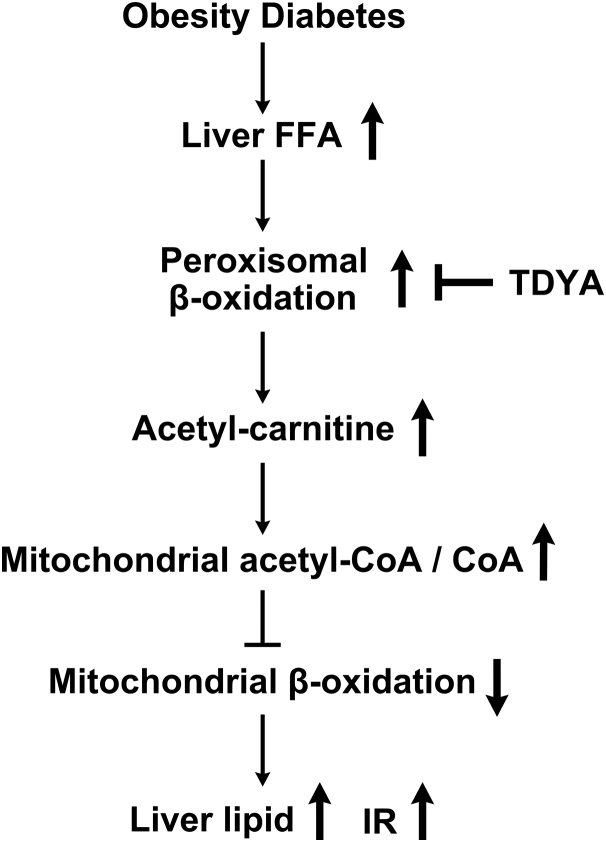
**Proposed mechanism by which peroxisomal β-oxidation causes suppression of mitochondrial FAO and lipid accumulation in the liver of obese or diabetic animals.** FAO, fatty acid oxidation.

Obesity has been known to induce lipid accumulation in liver, which might increase the risk of non-alcoholic steatohepatitis and IR. However, the mechanism by which dysregulated glucose and FAO causes suppression of mitochondrial FAO and lipid accumulation in liver is not fully demonstrated. The results revealed a novel mechanism by which suppression of peroxisomal β-oxidation enhances mitochondrial FAO in the liver of obese and diabetic animals, and a crosstalk between peroxisomal β-oxidation and mitochondrial FAO is established. The proposed mechanism was shown in Figure 8. Obesity or diabetes causes elevation in plasma free-fatty acids, which leads to increased hepatic uptake of fatty acids and induction of peroxisomal β-oxidation. Upregulation of peroxisomal β-oxidation results in increased generation of acetyl-CoA and acetyl-carnitine, and significantly elevated liver acetyl-CoA/CoA ratio, which causes suppression of mitochondrial β-oxidation ultimately results in hepatic steatosis in the obese mice.

Mitochondrial fatty acid β-oxidation is the major pathway for the metabolism of fatty acids and is essential for maintaining energy homeostasis in animals as well as human body (33); however, the control mechanism of mitochondrial FAO is complex and regulated by multiple factors. One of the most important control sites of mitochondrial FAO is CPT-1a, the rate-limiting enzyme in transporting fatty acyl-CoA into mitochondria (1, 2). Malonyl-CoA is identified to be a critical molecule in regulating the activity of CPT-1a, thereby controlling mitochondrial FAO. However, it should be noted that the biosynthesis of malonyl-CoA is stimulated by carbohydrate oxidation and suppressed by LC-CoA (1, 2, 34). Under the condition of diabetes, the supply of glucose is limited and liver LC-CoA increases significantly, which leads to the suppression of fatty acid synthesis and liver malonyl-CoA is depleted or not increased significantly; therefore, malonyl-CoA does not play a role in controlling mitochondrial FAO in the liver of diabetic or severe obese animals.

It is reported that mitochondrial FAO can be regulated by intramitochondrial NADH/NAD^+^ ratio, and elevation in NADH/NAD^+^ ratio might cause suppression of mitochondrial β-oxidation at 3-hydroxyacyl-CoA dehydrogenase step, leads to accumulation of 3-hydroxyacyl-CoA and 2-enoyl-CoA intermediates, and ultimately results in the suppression of ACDs and accumulation of LC-CoA (17, 35). However, it is also reported that regulation of mitochondrial FAO by NADH/NAD^+^ ratio is prominent only when this ratio is very high (16). Our previous study suggested that mitochondrial FAO was suppressed by succinate that generated from peroxisomal β-oxidation of endogenous dicarboxylic acids, which remarkably elevated mitochondrial NADH redox state and suppressed mitochondrial FAO by inducing accumulation of FAO intermediates (13).

Mitochondrial FAO can also be controlled by intramitochondrial acetyl-CoA/CoA ratio, and high acetyl-CoA/CoA ratio causes suppression of 3-ketoacyl-CoA thiolase and accumulation of 3-ketoacyl-CoA intermediate (14, 15, 16, 17), which is a strong inhibitor for ACDs, the rate-limiting enzyme in mitochondrial FAO (31, 32), thereby causing feedback suppression of mitochondrial β-oxidation. This study revealed that peroxisomal β-oxidation significantly elevated cellular acetyl-CoA/CoA ratio and caused diminished mitochondrial FAO and accumulation of LC-CoA and TAG in the liver of obese mice.

To determine the potential source that might cause elevation in mitochondrial acetyl-CoA/CoA ratio, it was reported that cytosolic acetyl-CoA can hardly be transported into mitochondria because mitochondrial CAT shows no significant activity towards acetyl-CoA outside the mitochondrion (36). Therefore, the elevation in acetyl-CoA within mitochondria is not due to increased cytosolic acetyl-CoA.

CAT is reported to be located on the inner membrane of mitochondria and catalyzes the transfer of acetyl groups from cytosolic acetyl-carnitine to acetyl-CoA within mitochondria (37). The content of acetyl-carnitine increased significantly in the liver of diabetic animals, which might be a potential source of mitochondrial acetyl-CoA. The activity of CAT was also upregulated in the diabetic animals, which greatly accelerated transformation of cytosolic acetyl-carnitine to mitochondrial acetyl-CoA. Therefore, increased generation of acetyl-carnitine might play a role in controlling mitochondrial through elevating mitochondrial acetyl-CoA/CoA ratio. In this study, the source of acetyl-carnitine was identified and the results showed that peroxisomal β-oxidation of fatty acids generated acetyl-carnitine and significantly increased liver acetyl-carnitine/carnitine ratio, which further caused increase in mitochondrial acetyl-CoA/CoA ratio and negatively regulated mitochondrial β-oxidation.

Peroxisomal β-oxidation is induced under the condition of high fat diet, fasting, diabetes, or hyperlipidemia drugs (10, 11). In recent years, more and more evidence suggested that peroxisomal FAO system might play a negative role in regulating mitochondrial fatty acid metabolism and inducing hepatic steatosis. Our previous study suggested that increased endogenous substrate flux through peroxisomal β-oxidation caused suppression of mitochondrial FAO by stimulating synthesis of malonyl-CoA, and specific suppression of peroxisomal β-oxidation stimulated mitochondrial β-oxidation and improved hepatic steatosis in rats fed a high fat diet (12, 26). Furthermore, it was reported that hepatic FAO increased significantly in ACOX1-/-*ob/ob* mice and mice with liver-specific knockout of ACOX1 (ACOX1-LKO), with lower level of hepatic acetyl-CoA and improved hepatic steatosis (38, 39), which are in well agreement with our results and support the proposed mechanism. Peroxisomal β-oxidation can also negatively regulate mitochondria β-oxidation and induce lipid accumulation in the liver of fasting animals through generating succinate and elevating intramitochondrial NADH/NAD^+^ ratio (13), while in the current study, liver succinate and 3-hydroxybutyrate/acetoacetate ratio were not altered significantly in *ob/ob* mice treated with TDYA or C22:1 (data not shown), suggesting that succinate might not play a role in regulating mitochondrial fatty acid metabolism and developing hepatic steatosis in obese and diabetic animals. It was reported that the acetyl-CoA derived from liver peroxisomal FAO inhibits autophagy and promotes hepatic steatosis via mTORC1 activation (39). This study reveals a novel mechanism by which peroxisomal β-oxidation regulates mitochondrial FAO in the liver of diabetic animals that is independent of malonyl-CoA or succinate and indicates that upregulation of peroxisomal FAO causes suppression of mitochondrial β-oxidation by elevating acetyl-CoA/CoA ratio and inducing accumulation of reaction intermediates.

Because of the complexity of the metabolic systems, the onset of hepatic steatosis might be caused by multiple factors. Mitochondrial dysfunction has been well accepted to play a critical role in inducing hepatic lipid accumulation and IR (27, 28, 29, 40). This study demonstrates a potential pathogenic mechanism by which induction of peroxisomal FAO causes suppression of mitochondrial β-oxidation and hepatic steatosis in animals; the results provide evidence that the diminished mitochondrial FAO and lipid accumulation in the liver of obese and diabetic animals are due, at least in part, to increased acetyl-carnitine generation in peroxisomes and elevation in mitochondrial acetyl-CoA level. It is well known that peroxisomal β-oxidation is induced in the liver of obese and diabetic rodents (18, 19, 20), which plays a role in the development of hepatic steatosis and IR. Regarding the situation in obese humans, there is evidence that peroxisomal FAO is also upregulated, as reflected by significant increase in peroxisome number and peroxisomal H_2_O_2_ level (a byproduct of peroxisomal β-oxidation) in the liver biopsies of obese humans (41, 42). Therefore, targeting peroxisomal β-oxidation might be a potential pathway in improving hepatic steatosis and IR as induced by obesity or diabetes.

## Experimental procedures

### Materials

Coenzyme A sodium salt, malonyl-CoA, acetyl-CoA, Percoll were purchased from Sigma. Erucic acid and TDYA were from Tokyo Chemical Industry. The CoA thioesters of erucic acid (C22:1-CoA) and TDYA (TDYA-CoA) were prepared according to the method as described previously (43).

### Animal studies

*ob/ob* obese mice (C57BL/6J genetic background) at the age of 8 weeks were purchased from Nanjing Model Animal Center, and C57BL/6J mice at the age of 8 weeks were from Slac Laboratory Animal Co Ltd C57BL/6J mice (N) were fed standard rodent diet (12% fat by calories). *ob/ob* mice were fed experimental rodent diet, with standard rodent diet containing either 5% oleic acid (*ob/ob* control) or erucic acid (*ob/ob*-C22:1). For the purpose of suppressing peroxisomal β-oxidation, TDYA at 100 mg/kg was administered by gavage to the *ob/ob* mice, once per day at 5 PM. All animals were housed in single cage with free access to food and water under controlled temperature (22 °C) and light (12 h of light and 12 h of dark). Blood glucose was determined from tail vein by a glucometer (Lifescan, Johnson and Johnson). The HOMA-IR index was calculated as described previously (44). Oral glucose tolerance test was performed on day 17; 1g glucose/kg was introduced intraperitoneally for each mouse, with blood samples collected at indicated time from tail vein and blood glucose was determined by a glucometer. On the day for scarification, the animals were deprived of food at 8 AM and sacrificed at 1 PM. Livers were removed quickly and stored in liquid nitrogen immediately. Histological analysis was performed according to the protocol as described previously (12). All the animal studies were approved by the Animal Care Committee of Hunan University of Science and Technology.

### Isolation of mitochondria and peroxisomes

Mitochondria were isolated by the method of differential centrifugation (45), and peroxisomes were purified by a Percoll gradient according to the methods as described previously (46, 47).

### Quantitative real time PCR

Total RNA was extracted from liver tissues with TRIzol reagent. RNA was reverse-transcribed with standard reagents (Applied Biosystems) using random primers. Complementary DNA was amplified in a 7500 Fast Real-time PCR System using 2×SYBR Green Supermix (Applied Biosystems). The used primers were: ACSL1,5'-TCCAAAAGGAAAGAGGCGGA -3′ (F) and 5′-TCCTCAGAAACGTCAGCACT-3′ (R); ABCD1, 5′- ATGAAGGAAGAGGAGCTGGT -3′ (F) and 5′- TGGAACATCTCGTACACCCT -3′ (R); L-BP, 5′- AAATACAGAGATACCAGAAGCCG-3′ (F) and 5′ - AAGAATCCCCAGTGTGACTTC -3′ (R); Thiolase, 5′- CCTGACATCATGGGCATCG-3′(F) and 5′-AGTCAGCCCTGCTTTCTGCA-3′(R); ACOX1, 5′- CGTTACGAGGTGGCTGTTAA-3′ (F) and 5′- GCATCCATTTCTCCTGCTGA -3′(R); MCACD, 5′- TCGGTGAAGGAGCAGGTTTC -3′ (F) and 5′- TTCGTGGCTTCGTCTAGAGC-3′(R); LCACD, 5′- GGAGCATGACATTTTCCGGG -3′ (F) and 5′- AGAGCAAGTCCCCACCAATG -3′(R); CAT, 5′- AACCTCCAACCACCGAAACA -3′ (F) and 5′- ACTTGCTACCACCACCATGT -3′ (R). mRNA expression levels normalized to 18S rRNA were expressed using the comparative delta CT method.

### Biochemical analysis

Plasma-free fatty acid concentration was determined using a colorimetric kit (Wako Pure Chemical Corporation). Plasma TAG was determined by commercial kit according to the manufacturer’s instructions (Wako). Plasma total ketone body was determined enzymatically (48). Plasma insulin was measured by a mouse insulin ELISA kit from Merck Millipore. Liver acetyl-CoA and free CoA were determined enzymatically according to the method as described previously (49). Liver acetyl-carnitine was assayed by the method of Pearson (50). Extraction of plasma fatty acids and measurement of hexacosanoic acid (C26:0) were performed according to the method as described previously (51). Ketone body synthesis by liver homogenate with hexanoate as a substrate was performed as described by McGarry (52). Mitochondria ACD activity was assayed by the method as described previously (53); isolated mitochondria (2 mg protein) and 20 μM palmitoyl-carnitine were used in the assay. Peroxisomal β-oxidation was assayed by acyl-CoA–dependent NAD^+^ reduction in the presence of KCN (54), with 100 μM C22:1-CoA as substrate. Liver LC-CoAs were extracted and determined by the method of Tubbs and Garland (49). Liver TAG were extracted by the method of Bligh and Dyer (55) and determined with a commercial kit (Wako). Liver hydrogen peroxide, citrate, and acetate were determined by commercial kits (Sigma). Liver malonyl-CoA was analyzed by HPLC as described previously (56). Liver thiobarbituric acid–reactive substances were assayed by a kit from Sigma. CAT activity was assayed as described before using 100 μM acetyl-carnitine (57).

### Statistical analysis

Data are presented as mean ± SEM. n = 8 for all the groups. The significance of differences was evaluated using Student's test by SPSS 18.0. *p* < 0.05 was considered statistically significant between paired groups.

## Data availability

All data are contained within the article.

## Conflict of interest

The authors declare that they have no conflicts of interest with the contents of this article.
